# Removal of Gross Artifacts of Transcranial Alternating Current Stimulation in Simultaneous EEG Monitoring †

**DOI:** 10.3390/s19010190

**Published:** 2019-01-07

**Authors:** Siddharth Kohli, Alexander J. Casson

**Affiliations:** School of Electrical and Electronic Engineering, The University of Manchester, Manchester M13 9PL, UK; siddharth.kohli@postgrad.manchester.ac.uk

**Keywords:** EEG, tACS, artifact removal, adaptive filter

## Abstract

Transcranial electrical stimulation is a widely used non-invasive brain stimulation approach. To date, EEG has been used to evaluate the effect of transcranial Direct Current Stimulation (tDCS) and transcranial Alternating Current Stimulation (tACS), but most studies have been limited to exploring changes in EEG before and after stimulation due to the presence of stimulation artifacts in the EEG data. This paper presents two different algorithms for removing the gross tACS artifact from simultaneous EEG recordings. These give different trade-offs in removal performance, in the amount of data required, and in their suitability for closed loop systems. Superposition of Moving Averages and Adaptive Filtering techniques are investigated, with significant emphasis on verification. We present head phantom testing results for controlled analysis, together with on-person EEG recordings in the time domain, frequency domain, and Event Related Potential (ERP) domain. The results show that EEG during tACS can be recovered free of large scale stimulation artifacts. Previous studies have not quantified the performance of the tACS artifact removal procedures, instead focusing on the removal of second order artifacts such as respiration related oscillations. We focus on the unresolved challenge of removing the first order stimulation artifact, presented with a new multi-stage validation strategy.

## 1. Introduction

Recent years have seen the development of non-invasive brain stimulation technologies. In particular, transcranial Electrical Stimulation (tES) is a relatively new technique with many applications as a therapeutic and investigative tool [1]. It operates by injecting small amounts of current into the scalp via rubber electrodes that are enclosed in saline soaked sponges and is available in several forms: transcranial Direct Current Stimulation (tDCS) applies constant current and is the most widely used; transcranial Alternating Current Stimulation (tACS) applies sinusoidal oscillating currents; transcranial Random Noise Stimulation (tRNS) applies randomly generated currents; and transcranial Pulsed Current Stimulation (tPCS) applies on/off tDCS periodically [2].

When paired with simultaneous neuroimaging these stimulation approaches offer the potential to make closed loop neuromodulation systems where the stimulation is tailored to the brain state of the subject at that particular point in time [3,4,5]. The effects of tES are highly variable between different subjects and studies [6,7], and it is thought that allowing brain state dependent stimulation paradigms may be a key step towards reducing this variability and understanding its causes. Focusing on tACS, the frequency and phase of the applied stimulation may be adjusted in real-time to deliberately match, or not match, the dominant EEG frequencies present.

While a number of fMRI+tES studies [8,9], and and magnetoencephalography (MEG)+tES studies [10,11], have been investigated, EEG (electroencephalography) is the more natural complement to tES as it is based upon recording voltages while tES injects currents [12,13]. EEG measures electrical activity which reflects temporal changes in the electrical state of neurons and represents the current flow, which is directly modulated when applying tES [1]. In addition, EEG has a very good temporal resolution allowing changes in the brain due to tES to be seen, essential for future closed loop systems. Although the EEG has a limited spatial resolution it is also readily portable for potential integration into future tES based therapeutics.

**Figure 1:**
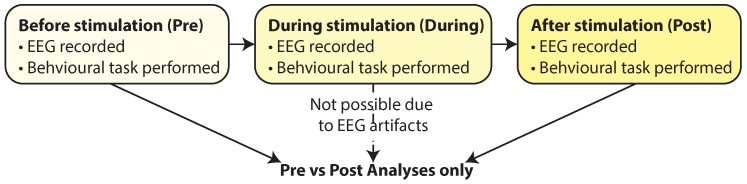
Most combined EEG+tES experimental protocols use only a comparison of EEG data before and after the stimulation due to the presence of stimulation artifacts in the EEG trace during stimulation. Figure originally reported in [12] (with copyright permission from IEEE).

However, simultaneous imaging via EEG and stimulation via tES is not possible at present due to the presence of stimulation artifacts in the EEG trace which occur during tES and prohibit the direct analysis of simultaneously collected EEG data. For example, Ref. [14,15] investigated the effect of tACS by recording EEG activity, but only after the tACS stimulation had been stopped. Their results displayed an elevation of the alpha band post stimulus. This is characteristic of most work looking at recording EEG activity with tES which adopt a model of only comparing data collected before and after stimulation, see Figure 1. Early work on closed loop tACS+EEG systems has similarly adjusted the frequency/phase of the applied stimulation based on an on/off protocol, measuring the EEG properties while the tACS is off, and then forecasting the properties into the future for adjustment of the stimulation settings while the tACS is on [16].

**Figure 2:**
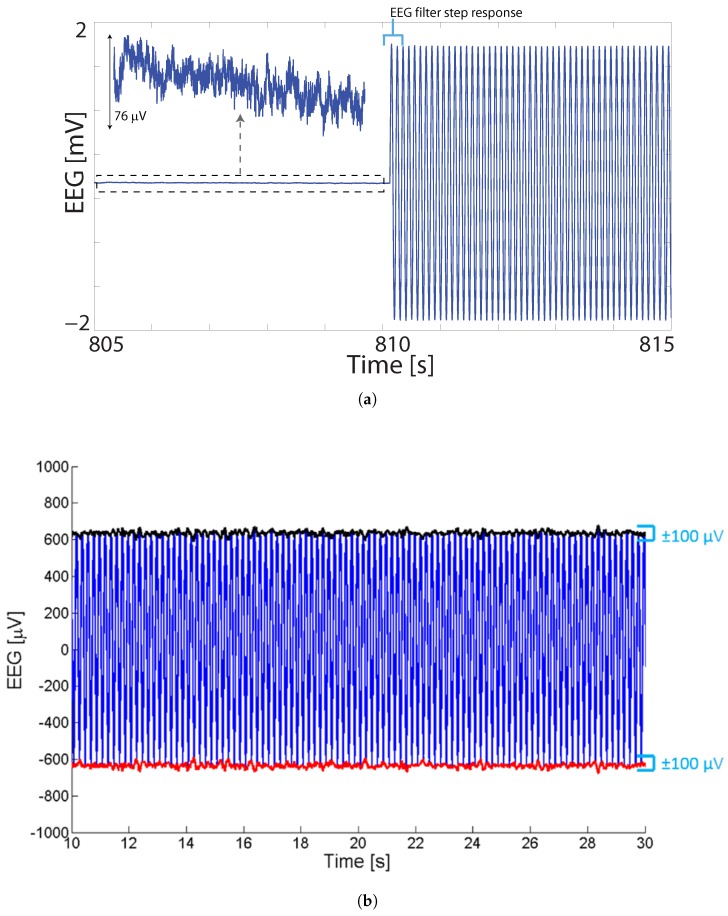
EEG data recorded during tACS stimulation. (**a**) Stimulation begins at 810 s at which point all recorded data are dominated by a large sinusoidal artifact. Figure originally reported in [12] (with copyright permission from IEEE). (**b**) Raw collected EEG+tACS data with no band limiting filters applied. Ongoing artifact is not a pure sinusoid at the simulation frequency, but has an approximately 100 μV ripple present.

During tDCS, artifacts are presented as low frequency noise which have been isolated using Independent Component Analysis (ICA) [17,18]. In contrast, in tACS the gross artifact manifests as a sinusoidal signal at the stimulation frequency as shown in Figure 2a. With one stimulation source, approximately the same tACS artifact is present at all of the EEG electrodes and so is significantly attenuated by the Common Mode Rejection of the EEG amplifier (which is normally used to suppress 50/60 Hz mains noise collected by all of the electrodes). The common mode rejection ratio of our EEG amplifier from Neuroelectrics (Barcelona, Spain) is −115 dB, and so the interferer present at the stimulator source could be up to 115 dB larger than that shown in Figure 2a. Even after the common mode rejection of the interferer the tACS artifact is still much larger than the true EEG signal to be recorded. In our measurements, at stimulation amplitudes of 250 μA and 1 mA, the Signal-to-Noise Ratio (SNR), i.e., the ratio between the root-mean-squares of true EEG data and tACS artifact, was found to be −23 dB and −33 dB respectively.

These are substantial artifacts, and as tACS is typically applied overlapping with the EEG frequencies of interest (5–40 Hz) it is not possible to remove the artifact using a simple notch filter at the stimulation frequency. As the bandwidth of the notch filter will inevitably be wider than the single frequency of the stimulation this would also remove a large amount of EEG information. Moreover, as shown in Figure 2b the gross artifact is not a pure sinusoid. The true amplitude of artifacts at each individual electrode is unknown and will vary for different electrodes since some energy will be lost as the injected current passes through the conductive layers of the skull and the brain. The impedance of the electrodes are never truly constant and thus multiple factors (such as electrodes drying, blood circulation under electrodes, muscle movements and similar) can result in changes in impedance. This will directly result in a change of stimulator’s output since it is trying to maintain a constant current and will adjust the output voltage based on impedance and thereby modulating the artifact itself. The result is the 100 μV variability shown in Figure 2b which would still dominate over true EEG even if the sinusoidal interferer was fully removed.

Similar characterization of the tACS artifact was presented by [19], which discussed the modulation of the tACS artifact in EEG and MEG recordings due to ongoing processes such as respiration and heart beats. Investigations into these have suggested that the non-linear artifacts are non-stationary, occurring when there is (say) a heart beat and are only present for a short duration after that rather than all of the time. They occur at the mixing frequency between the stimulator and the heart rate and at harmonics of these. Other works discussing these non-linear artifacts have suggested that they originate at the stimulator rather than at the electrode [20]. In this case, the artifacts occur when the stimulator is adjusting its output to maintain a constant current.

In this article we present two techniques for removing the gross tACS artifacts, as seen in Figure 2 as the sinusoidal signal and 100 μV ripple, from simultaneously collected EEG data and do not consider these non-linear and secondary artifacts. Instead we give particular attention to the verification process used in order to assess the level of artifact removal present. There is much debate as to *how good* the artifact removed EEG needs to be in order for it to be usable, with this discussed in detail in [19,20], and this debate is likely to be ongoing. We have used a multi-stage and comprehensive testing strategy to not rely on any one set of experiential evidence, but to use several separate analyses to build confidence in our artifact removal process and show that the EEG it produces contains true brain related information. This is in contrast to other methodologies for verifying EEG/stimulation artifact removal such as the one proposed in [21] which only visually inspected time and spectral domain data, and that in [22] which visually inspected spectral data and compared one EEG descriptive statistic. Our methodology combines the use of phantom head models and on-person testing, detecting alpha activity in the time and frequency domains, EEG descriptive statistics, and detecting Event Related Potentials (ERPs) during tACS. A preliminary version of this paper was published as brief conference papers [12,13].

## 2. Methods

### 2.1. Overview

We present two techniques in order to explore the different trade-offs available in terms of artifact removal performance, the amount of data required for the method to work, and their suitability for closed loop systems. In particular, we consider techniques which are:

- Independent of the number of EEG channels available, allowing for quicker experimental setups, potentially with low EEG channel counts, which may be critical for clinical applications and when working with vulnerable subjects.
- Time-localized/adaptive so that the removal process adapts with different contact impedances to minimize the induction of secondary or non-linear artifacts.
- Suitable for real-time implementation in order to allow brain state adaptive stimulation protocols to be developed, dynamically matching the tACS parameters (e.g., phase, frequency) to that of the underlying EEG.
- Removed from each individual record, not as an average removal when collecting data across multiple people or trials. This is particularly important if EEG+tACS is to find a role in closed loop neuromodulation applications where results from multiple runs cannot be averaged together if the system is to operate in real-time.

The Superposition of Moving Averages (SMA) method was introduced in [12] to provide a low computational complexity, channel count independent method of artifact removal. This uses the collected EEG data to build a time-localized template of the artifact which is currently present, subtracting this from the currently collected data. In contrast the Adaptive Filter (AF) technique is to our knowledge the first application of adaptive filters to the tACS artifact removal problem. Adaptive filters are commonly used to remove known, yet varying, sources of noise from systems such as noise canceling headphones (see for example [23,24]). During tACS, the injected current is in principle *known* and can be recorded, thus making parametric signal processing very applicable.

### 2.2. Superposition of Moving Averages (SMA)

**Figure 3:**
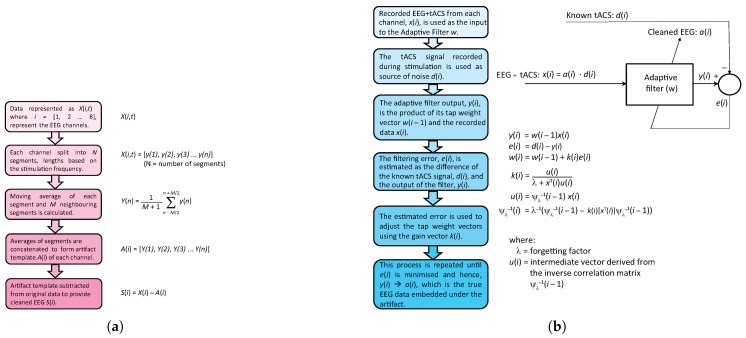
Artifact removal algorithms. (**a**) Superposition of Moving Averages (SMA): A time localized artifact template is generated for each channel and subtracted from the recorded data. Figure originally reported in [12] (with copyright permission from IEEE). (**b**) Adaptive Filtering (AF): A recorded version of the tACS output is used to dynamically set the artifact removal filter coefficients.

The SMA approach was introduced by the authors in [12], by noting that the periodic aspect of the tACS artifact is, in some regards, similar to the periodic artifacts seen in EEG data recorded during functional Magnetic Resonance Imaging (fMRI). SMA thus applies similar techniques to those in fMRI studies previously (for example [25,26]) using a moving window to build a time localised estimate of a current artifact template and subtracting this from the raw EEG data. This methodology is illustrated in Figure 3a.

Let the EEG data be represented as a matrix $X_{I\times T}$, where *I* is the total number of channels and *T* is the number of time samples. Data from each individual channel *i* at time *t* X(i,t) is first split into *N* non-overlapping segments such that the length of each segment matches the period of the tACS stimulation frequency. If the period of stimulation cannot be split into segments with an integer number of samples the segment length is set to be as small as possible while also being periodic and having an integer number of samples. For example, the segment length for one period of 40 Hz stimulation sampled at 500 Hz is 12.5 samples, so 25 samples, two periods of the stimulation frequency, are used.

Then each segment, y(i,n), where n=[1,2...N], and its *M* neighboring segments are central moving averaged to create a time localised artifact template A(i,n) for each corresponding segment y(i,n). This artifact template is bespoke for each channel, and evolves over time with span determined by *M* to adapt to changing conditions (such as changing impedances) during the tACS stimulation. The artifact template A(i,n) is then subtracted from the data and the resulting signal, S(i,t), represents the estimate of true EEG data during stimulation. The artifact template is specific for each EEG channel, thus, this methodology is independent for each EEG channel, making it more suitable for use in out-of-lab conditions with low (and even single) channel counts. SMA has been implemented in the open source toolbox [27], which showed that it acts as a comb filter with the frequencies at the fundamental tACS frequency and harmonics removed.

For this study *M* is selected to be 5% of *N* (the total number of segmented epochs) based upon data collected for our preliminary experiments in [12]. The study in [12] used a different data set to that used here and so there is no *in-sample testing* using the same data set to both optimize the algorithm parameters and to then evaluate the performance. Such a testing approach could artificially increase the apparent performance of the artifact removal approach. Previously M=5% showed to give good performance and a suitable trade-off between the number of averages taken and the time localization of the artifact template within a one minute stimulation period.

### 2.3. Adaptive Filtering (AF)

Adaptive filtering is a parametric time varying filtering approach [28]. As opposed to conventional filters which have fixed filter coefficients the AF filter coefficients are varied depending on the accuracy of previous filtering iterations using an optimization algorithm and error cost function derived from the input *noisy* signal (EEG+tACS) and an estimate of the interfering source (tACS) [28]. This approach has been widely used for noise-canceling headphones where the noise signal (wanted audio+background noise) is separated from an estimate of the interference (background noise) recorded using a microphone [29]. If an estimate of the tACS artifact is known the EEG+tACS artifact system can be thought of as equivalent to this. An overview of our application of this principle is shown in Figure 3b.

It is possible to record the output of some tES stimulators, including the one used in this study, as an analogue voltage signal which can then be used for the adaptive filter as the *known noise* (d(i)). We adjust the amplitude of this recorded tACS signal by multiplying by the root mean square of the EEG data at a given channel. This forms one input (d(i)) to the AF, with the second input (x(i)) being the EEG recording, which is a mixture of the true EEG (a(i)) and the tACS signal (d(i)).

For formulating the adaptive filter, several different methods are available and from preliminary work we selected the *RLS* algorithm [28]. This method utilities a weighting factor to minimize the error estimates which can be manipulated by adjusting the value of a constant called the forgetting factor, λ (a positive constant in the range of 0 to 1). By selecting a small value for the forgetting factor the cost function puts more emphasis on recent values of error estimates (forgets the past) whereas a value closer to 1 increases the *memory* of the algorithm and hence it includes older estimates when determining its coefficients. A system with higher memory intuitively fits the case of our setup where the tACS signal is periodic and thus deterministic (not random) and consequently produces a more stable output when fed into an RLS filter with a high forgetting factor. RLS adaptive filters are also known for having a much faster convergence (stabilization of the output at startup) at a cost of higher computational complexity, which is expected when the algorithm is required to retain previous estimates [28].

In our setup the recorded EEG signal during stimulation for each channel is individually sent to the adaptive filter. Thus similar to SMA, the adaptive filtering is independent of the number of EEG channels and is time varying and thus able to adjust based on the changes in the recorded artifact signal over time.

### 2.4. Phantom Head Creation

**Figure 4:**
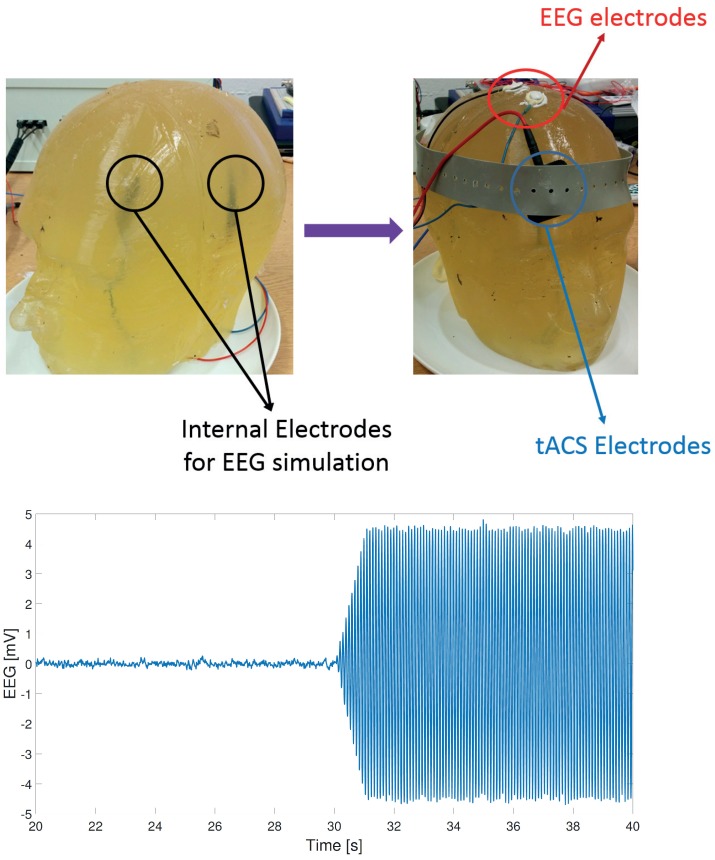
Gelatin phantom head model. (**Top**) internal electrodes for generating EEG signal, with tACS and EEG electrodes placed on the surface. (**Bottom**) Recorded EEG after 10 Hz, 1 mA tACS starts at time 30 s. Here a 1 s ramp up is included in the stimulation settings.

To allow controlled testing of the artifact removal process we created a gelatin phantom head model which would allow *known* EEG signals to be played out and recorded, while tACS stimulation was also applied to the model. This builds upon the work from [13,30], where a gelatin head model has been used for electrode and motion artifact characterization. We follow a similar procedure, now also applying tACS to the model. The setup is shown in Figure 4.

15% gelatin was poured into a head shaped mold, created by 3D printing the inverse of a head scan of a human dummy used for automotive testing [31]. During pouring of the gelatin two electrodes were placed inside the head mold, with wires extending outside of the model such that they could be connected to an external bi-phasic current generator (Digitimer, DS4). The assembly was then refrigerated for 8 hours, after which the shell was removed leaving behind the gelatin phantom head model. Electrical properties of ballistic grade gelatin are reported in [32]. EEG activity on the surface of the model was simulated by connecting the bi-phasic current generator to an arbitrary waveform generator which could output pre-recorded EEG data collected from a human subject. This injected *EEG shaped* currents into the phantom head, which manifest as EEG voltages on the surface of the phantom and can be recorded using standard EEG acquisition hardware. We recorded a single channel of EEG for algorithm validation purposes (as only a single source is present in our model) with reference (Cz), ground (FCz) and recording (P4) electrodes placed on surface of the phantom head. All other EEG factors are identical to those used for on-person testing.

Similarly tACS electrodes were placed on the head model with the same montage as in the on-person experiments. Stimulation settings again matched. To create a realistic combined signal of simulated EEG and applied tACS, the pre-recorded EEG amplitude was scaled so that the SNR of the recorded EEG+tACS matched that of EEG+tACS data recorded during real-life stimulations. This was calculated by measuring the EEG amplitudes before and during stimulation for all stimulation protocols for all subjects. The average SNR for the 250 μA and 1 mA stimulation amplitudes was found to be −23 dB and −33 dB respectively, with no difference in SNR between stimulation frequencies. An example of the collected EEG+tACS signal from the phantom head is shown in Figure 4.

### 2.5. Experimental Procedures

#### 2.5.1. Experimental Protocol

**Figure 5:**
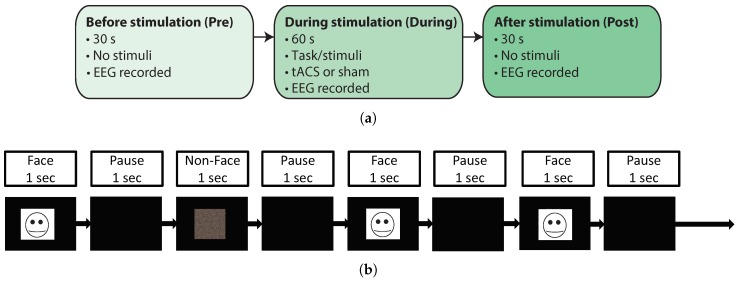
Overview of experimental protocols. (**a**) The *Pre* and *Post* stages are 30 s long with no stimuli presented. The *During* stage is a minute long task/stimuli which is presented during tACS stimulation or during a sham where the same task is performed but no tACS is applied. (**b**) Visual evoked response task protocol. Face and non-face images were shown in a randomized order for 1 s at a time followed by a 1 s pause with a blank screen. This figure shows an example of a randomized face, non-face, sequence. A picture of a famous person was used in the experiment, with the actual image not reproducible here due to copyright constraints.

Two different EEG+tACS experiments were performed in order to demonstrate the operation of the artifact removal algorithms in different situations. Both experiments followed the same design, shown in Figure 5a. The experimental tasks/stimuli and tACS stimulation only occurred in the 1 min long *During* stage, while in the *Pre* and *Post* stages, both 30 s long, subjects were asked to relax and do nothing so a baseline EEG recording could be established for comparison, and to allow a minimum gap between stimulations. The stimulation duration was set to a short duration of 1 min to avoid inducing long-term changes in the underlying brain state so that descriptive statistics could be applied and compared between the three experiment stages.

To investigate *free-running* background EEG recovery during tACS, we firstly collected alpha EEG activity. In the *During* stage participants were asked to keep their eyes open for the first 30 s and closed for the next 30 s, with directions given verbally. This allowed for spontaneous alpha activity to be detected at the point when the subjects closed their eyes. This was intended to give a known and sustained natural brain response that could be observed and used as a measure of performance of artifact removal. There were 8 repetitions of this protocol, two sham cases where no stimulation was applied and one for each of the 6 different tACS stimulation conditions (3 frequencies at 2 amplitudes, discussed below).

To investigate evoked EEG response recovery during tACS, the second protocol presented a visual face recognition task designed to produce P100, N170, and P300 evoked responses [33], which give a known, and very low amplitude, EEG component to recover. Participants were asked to look at images of celebrity faces to trigger recognition and also a *non* image which was made as a pixelated scramble of the celebrity face images, as shown in Figure 5b. In the *During* stage there were 30 different stimuli presented (15 face and 15 non-face stimuli) in a randomized order. In each run an image would appear on the screen for 1 s and there was a 1 s pause between each stimuli where the screen was blank. The protocol was repeated 12 times for each participant, with 6 sham and 6 stimulation cases.

#### 2.5.2. tACS Stimulation

For both the phantom head and on-person tests tACS was applied using two rubber electrodes placed in saline soaked sponges on FP2 and P3, with these electrode positions selected to mimic previously used montages in experiments looking at effects of parietal stimulation in working memory which implement a contra-lateral frontal-parietal tACS montage, for example [34]. The stimulation current was delivered using an isolated, battery-operated stimulator (DC–stimulator plus, Neuroconn, Germany), with the *signal out* option available to record the delivered signal for use with the adaptive filter.

To demonstrate artifact removal in a range of stimulation cases three different frequencies (5, 10 and 40 Hz) were used, each at amplitudes of 250 μA and 1 mA peak-to-peak which correspond to SNRs of −23 dB and −33 dB respectively. These were selected to be representative of high and low current stimulation values used in the wider tACS literature. The stimulation duration was 60 s for all 6 different conditions with a 1 s fade in and out of the stimulation amplitude at the start and end of stimulation. The stimulation frequencies of 5, 10 and 40 Hz were used to cover a wide range of EEG bands, and particularly 5 and 10 Hz stimulations were of interest due to their overlap (or near-overlap) with common cortical frequencies allowing us to investigate the feasibility of acquiring EEG during tACS, even with overlapping frequencies of interest.

tACS stimulation durations were deliberately kept short (1 min) in order to prevent lasting entrainment of neural oscillations such that brain changes due to stimulation are minimized. Our aim in this paper is to introduce as few brain changes as possible to allow EEG before, during and after stimulation to be compared for the presence of artifacts. To our knowledge no behavioral changes due to tACS have been reported for tACS stimulation durations less than 10 min, and we see no enhancement at any of the tACS stimulation frequencies post stimuli, implying success in not inducing brain changes with the short stimulation. Once the performance of the artifact removal has been established, in future studies we can target the behavioral effects of tACS and investigate the electrophysiological effect of tACS during stimulation.

#### 2.5.3. EEG Data Collection

EEG was recorded during tACS stimulation and for 30 s prior to and post stimulation. EEG data was acquired at a sampling frequency of 500 Hz using an 8 channel wireless EEG device (Enobio, Neuroelectrics, Barcelona, Spain).

Electrodes were placed at Fp1, F3, C3, C4, P4, PO7 and PO8 with the reference and ground electrodes placed next to each other at Cz. Electrodes were placed directly on the scalp, or head model, using an adhesive EEG paste (EC2 Electrode Cream, Grass Technologies, Pleasanton, CA, USA).

This EEG montage is designed to allow recording of EEG data without saturation of the amplifiers due to the tACS artifacts. The symmetric setup with placement of the reference on the mid-line at Cz makes most of the tACS interference signal appear as a common mode component (the same interference is present at all electrodes). The bulk of the interference is thus intrinsically rejected by the common mode rejection of the EEG amplifier which is 115 dB, leaving a much smaller (<10 mV) differential mode tACS interference (SNR −23 dB–−33 dB) to be removed by the algorithms proposed here. If this procedure is not followed, the full tACS interference of approximately 10 V (1 mA injected current through a 10 kΩ electrode impedance) saturates the EEG amplifier preventing the recording of any valid signals.

#### 2.5.4. Participants

We recruited 5 participants, 3 male and 2 female, aged 21–26. This number of participants was selected to be in-line with other EEG artifact removal works: Noury, et al. [19] used 5 subjects, Kim, et al. [35] used 12 subjects, Chowdhury, et al. [22] used 2 subjects, Baxter, et al. [17] used 5 subjects, Roy, et al. [18] used 8 subjects. Importantly, we highlight here that our aim is not to present (or imply to present) a behavioral result where averaging across a larger number of different participants is very significant. We present the technical results of the artifact removal process, and for this averaging across multiple subjects is not desirable as the artifact is either removed, or not, on an individual record-by-record bases.

All experiments with human subjects were conducted between 11 a.m. and 1 p.m. on a weekday to minimize baseline EEG variances and subjects were allowed to take breaks in between experiment runs to prevent fatigue. All procedures used in this study were reviewed and approved by the University of Manchester Research Ethics Committee, and all subjects gave informed consent before participating. The experiments were performed in accordance with relevant guidelines and regulations.

### 2.6. EEG Analysis Methods

A multi-step validation procedure was used to verify the data using a phantom head, time domain analysis, frequency domain analysis, and ERP domain analysis. The aim is not to rely on any one validation method, but to build an overall picture of the level of artifact removal. All recorded data was analyzed offline in Matlab (Mathworks, Natick, MA, USA). Prior to analysis the data was filtered using 3rd order low pass and high pass Butterworth filters with cut-offs at 50 and 3 Hz respectively.

#### 2.6.1. Phantom Head EEG Data

The SMA and AF algorithms were applied to the recorded EEG+tACS data. The use of the phantom head allowed for a direct comparison between the *EEG* played in at a particular point in time, and the recovered signal after artifact removal. To quantify this comparison, the SNR of the reconstruction has been calculated where the *signal* is the Root-Mean-Squared (RMS) value of the inputted EEG trace, and the *noise* is the RMS of the difference between the inputted EEG trace and the reconstructed trace after artifact removal. Finally the Power Spectral Density (PSD) of the inputted EEG data is also calculated. Data was split into 1 s long epochs with a 0.1 s overlap, then each epoch windowed using a Hamming window. The periodograms (calculated with a $2^{15}$ point FFT) of these epochs averaged to obtain the PSD estimate. For inputting to the phantom data, on-person data from sham stimulations for the free running EEG task were used which could be split into *eyes open* and *eyes closed* parts.

#### 2.6.2. Time Domain: Visual Inspection of Data

In the time domain, visual inspection of the artifact removed signals was used to verify the emergence of alpha frequency patterns when the eyes were closed and the subject was in a resting state. Note that the data to be compared necessarily comes from different recordings (one during tACS, one during sham) and so the precise data will not be identical.

#### 2.6.3. Time Domain: Descriptive Statistics

Using a similar technique to that applied to assess the performance of artifact removal in EEG data during fMRI ([22,36]), descriptive statistics of the data collected during sham and and artifact removed tACS stimulation were derived and compared to determine whether there was a statistical difference between true EEG and artifact removed EEG. As measures we used the complexity, kurtosis, Root-Mean-Squared (RMS) amplitude, and zero-crossings to represent statistical properties of EEG data, selecting these metrics as: Kurtosis is used in [35]; RMS as in [22]; complexity, used as a measure of signal entropy which is expected to increase in the presence of artifacts with no strong structure present [37], although entropy could decrease if a large artifact was, say, purely sinusoidal; and zero-crossings to give a time domain estimate of the frequency content which will change if residual artifacts at the stimulation frequency are present.

These statistics are found for each 10 s non-overlapping window of EEG data, with the window size determined from a sensitivity analysis checking the accuracy of each descriptive measure with window size. Although sham and stimulation EEG data are from different recording cases and the statistics are not expected to be identical, our stimulations are deliberately short in duration and we do not expect differences in the brain state to be caused by tACS. The assumption is that the descriptive statistics of EEG data containing residual tACS artifacts would be different from those of sham conditions.

This is tested for statistical significance by a one way ANOVA (p<0.05) between sham and stimulation conditions for each measure for all subjects. Subsequently, a multiple comparisons test using scheffe’s method [38] was applied to determine the *p*-values, estimated mean differences and confidence intervals (95% confidence level). Thus if the gross tACS artifact is correctly removed the ANOVA null hypothesis should be not rejected. This is in contrast to the standard ANOVA formulation where the expectation is to reject the null hypothesis. As a result this test does not the prove descriptive statistics come from the same population, only that the collected data does not support the presence of residual artifacts which may cause a difference in the descriptive statistics. All data was tested for normality using the Kolmogorov-Smirnov test (p=0.01) before applying the ANOVA.

#### 2.6.4. Frequency Domain: Visual Inspection

Frequency domain analysis was performed on the free running (alpha) EEG data by calculating the PSD of the EEG using Welsh spectrograms, using the same settings as for the phantom head given above. As with the time domain analysis the PSDs between artifact removed EEG+tACS data and the sham are expected to be non-identical, but highly similar.

#### 2.6.5. Frequency Domain: Individual Alpha Frequency Extraction

To illustrate the potential for individual EEG data responsive stimulations the PSD data was used to extract the Individual Alpha Frequency (IAF) for each protocol (or section of protocol). This is taken as the frequency with the peak PSD value in the alpha range (8–12 Hz), and the ability to extract it during tACS shows how the stimulation frequency could potentially be dynamically adapted to match, or deliberately not match, the underlying brain state.

#### 2.6.6. ERP Domain: N170 Detection

Using the face task data ERPs were extracted to show that very low amplitude (<10μV) EEG components were correctly present in the artifact cleaned data. To extract responses the data for the visual ERP task was split into 1 s long sections corresponding to the duration for which each stimuli was presented. An ERP detection algorithm identified successful trials for both face and non-face stimuli by identifying the P100, N170 and P300 peaks, searching for the 3 peaks in the EEG data after each visual stimuli. For a successful trial these peaks were required to be greater than 0.5 times the mean absolute deviation of the baseline EEG data collected during the *pre* stage of each protocol. If all three peaks were detected, the ERP was accepted, otherwise it was discarded. The resulting trials were then averaged together to allow the ERPs to be plotted.

## 3. Results

### 3.1. Phantom Head EEG Data

**Figure 6:**
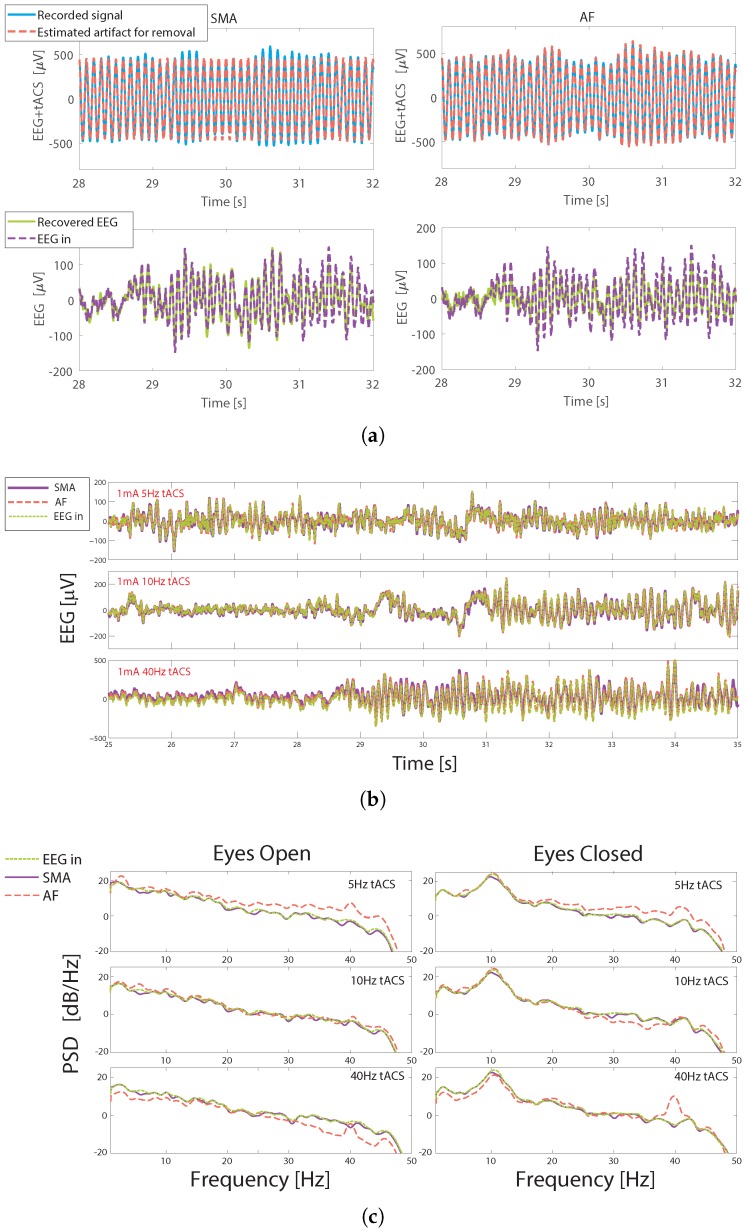
Illustrations of artifact removal from the phantom head. (**a**) The raw recorded signals and the artifacts which are estimated from these by the two algorithms. The recovered EEG signal compared to the one inputted to the phantom head is also shown. Traces are for a 10 Hz, 0.25 mA stimulation. (**b**) Example reconstructions for different tACS frequencies. All stimulation amplitudes are 1 mA. For both the SMA and AF approaches the recovered EEG signal visually closely matches that which is inputted into the head model. (**c**) Frequency domain representation. PSD at 0.25 mA stimulation, with the inputted EEG data split into *eyes open* and *eyes closed* periods.

The process of artifact removal on the phantom head is illustrated in Figure 6. Figure 6a shows the recorded EEG+tACS signal, and the recovered and *known* EEG after both artifact removal methods for a 10 Hz, 0.25 mA stimulation. It can be seen that both the SMA and AF techniques produce estimates of the artifact present which follows the gross tACS artifact. When this artifact estimation is subtracted from the recorded signal, reconstructions of the EEG data are seen. The recovered EEG signals are shown in Figure 6b for three different tACS frequencies, all with 1 mA amplitude. In all cases a very close match is seen with the inputted EEG. Both the SMA and AF artifact removal approaches remove the gross tACS artifact, allowing traces which closely follow the input EEG trace to be observed. The same results are illustrated in the frequency domain in Figure 6c. These show a residual peak at 40 Hz, particularly after AF artifact removal, which is also seen in the on-person tests discussed below. The peak in the alpha range in the eyes closed case is clearly seen, with this being present after both artifact removal methods.

**Figure 7:**
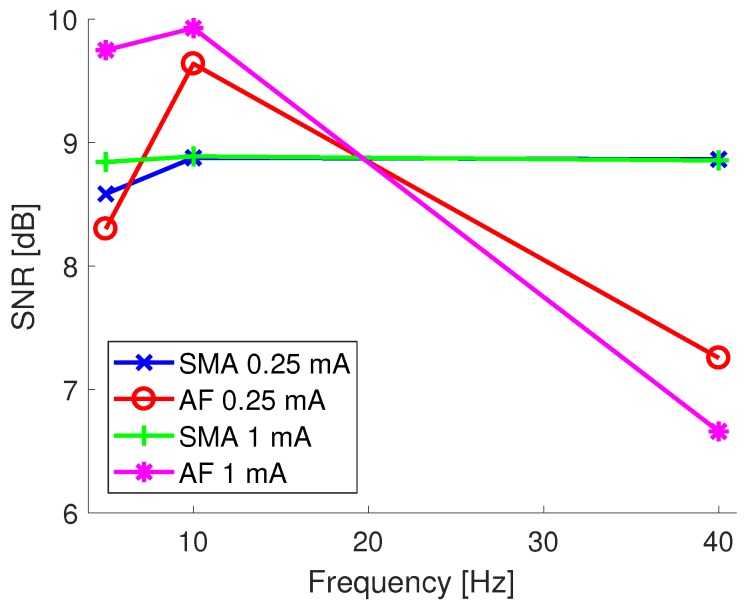
SNR between inputted and reconstructed EEG data collected using the phantom head after tACS artifact removal at different tACS stimulation settings.

The performance of the techniques across all of the tACS stimulation settings is quantified in Figure 7 via the SNR between the inputted and the artifact removed EEG signals, with this also showing the performance at different stimulation amplitudes. For the SMA approach the performance is very similar for the two different input tACS amplitudes, with the performance at 5 Hz and 0.25 mA stimulations being slightly worse. In comparison, the performance of the AF approach is much more dependent on frequency, with the recovered SNR reducing for 40 Hz stimulations. This effect is also seen in the on-person tests, and is discussed below. In all cases the SNR is above 6 dB. When assessed via the correlation coefficient between the different traces the worst case was 0.86 for the AF method when applied to a 5 Hz, 1 mA stimulation. The SMA method consistently outperformed the AF approach, with its worst case correlation being 0.95. In comparison the average correlation coefficients between EEG data and the data without artifact removal applied was less than 0.03 for all conditions.

### 3.2. Time Domain: Visual Inspection of Data

**Figure 8:**
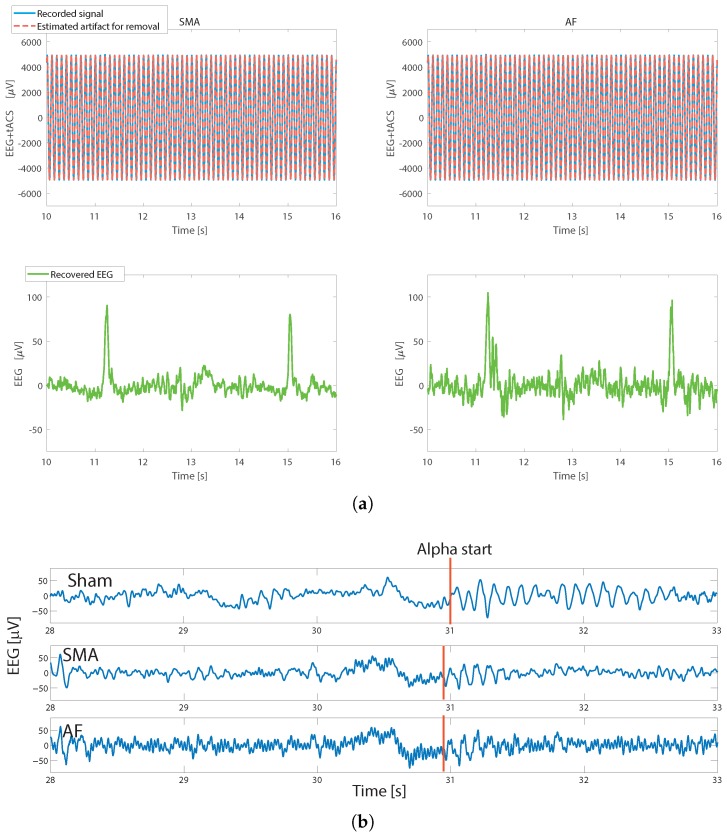
Time domain EEG after artifact removal. (**a**) The measured artifacts using the two algorithms and the recovered EEG during an eye blink. 10 Hz, 0.25 mA stimulation. (**b**) EEG data during the alpha task protocol, at PO8, for a single subject showing sham and artifact removed data with tACS at 40 Hz, 1 mA amplitude. Eyes are closed at the 30 s mark. Then bursts of alpha are seen for both sham and stimulation using all three artifact removal approaches. Note that the sham and stimulation are different trial runs and thus the EEG trace for sham and the other figures are not expected to be identical.

For on-person data the artifact removal performance is illustrated in Figure 8. Figure 8a is the on-person equivalent of Figure 6a, using the same stimulation settings, and showing the raw recorded signal, the estimated artifact, and the recovered EEG. This is shown during an eye blink to give an easily recognizable signal to observe.

Figure 8b then shows the EEG for one subject during 40 Hz, 1 mA stimulation. This is indented to give an example of the artifact removed EEG data, and to show that the data are removed from each individual record. For the practicalities of on-person recording where only one stimulation setting can be used at a time note that all three traces are separate experimental runs and so the recovered signals are not expected to be identical. Nevertheless, in all cases the subject closes their eyes at approximately the 30 s mark, and in both the sham and artifact removed EEG data a burst of alpha activity can be easily identified. The AF case clearly contained more high frequency residual artifact than the sham or SMA cases. Compared to the sham, in this case the initial alpha burst only lasted for approximately 0.5 s compared to 2 s in the sham case.

### 3.3. Time Domain: Descriptive Statistics

To quantify the artifact removal performance, and summarize the performance across all runs and subjects, Table 1 gives the descriptive statistics (complexity, kurtosis, RMS, zero-crossings) of the artifact removed EEG data and EEG data during sham stimulation for the free running EEG data. The ANOVA and multiple comparisons test accept the null hypothesis, which could suggest that no residual artifacts with different descriptive statistics are present. It suggests that the descriptive statistics of the EEG are similar, regardless of whether tACS was applied or not. This is further demonstrated by the majority of high *p*-values (0.9–1) for comparison of sham and stimulation data. The Kolmogorov-Smirnov test showed that values of the descriptive statistics satisfied normal distributions, apart from the Kurtosis descriptive statistic from the adaptive filter. As ANOVAs are relatively robust to deviations from normality for consistency we kept the same analysis method for the Kurtosis descriptive statistic rather than switching to a non-parametric test for this one comparison.

### 3.4. Frequency Domain: Visual Inspection

**Figure 9:**
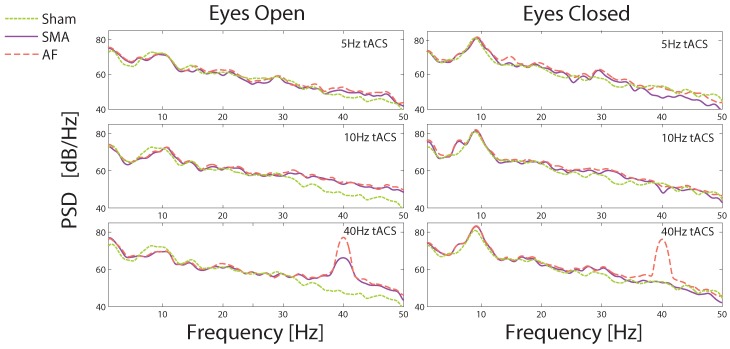
Power Spectral Density (PSD) data, at PO8, during the alpha task protocol for a single subject with stimulation at 5 Hz (top), 10 Hz (middle) and 40 Hz (bottom), 250 μA amplitude. The protocol is split into 2 sections, Eyes Open (left) and Eyes Closed (right). An increase in alpha activity (8–12) Hz is seen when the eyes are closed in all cases.

The artifact removal performance is illustrated in the frequency domain in Figure 9, which again shows data from a single subject to demonstrate the potential for individually data responsive stimulation protocols. PSD data are shown during 5 Hz, 10 Hz and 40 Hz tACS during the 30 s when the eyes were open, and during the 30 s when the eyes were closed. All of the eyes closed cases show a substantial increase in the alpha band power, as would be expected, in sham data and in true EEG data after tACS artifact removal. This is despite the fact that for 5 and 10 Hz stimulations the stimulation frequency overlaps, or near-overlaps, with the EEG frequency.

In the other frequency bands there is a close correspondence between the EEG powers in the artifact removed data and the sham data, again indicating that there are no residual tACS artifacts introducing distortions at particular frequencies, apart from in the 40 Hz stimulation case. Here a large peak at 40 Hz for the eyes open case was present with both methods, but lower than the dominant alpha rhythm which allowed the alpha activity to be seen in the time domain as in Figure 8. Nevertheless this indicates the presence of a residual artifact in the reconstructed EEG data. This peak was not seen in the eyes closed case (40 Hz stim) when using the SMA technique, which again is reflected in Figure 8 where high frequency artifacts are present after AF but not SMA artifact removal. The implications of this peak will be considered in the discussion section.

### 3.5. Frequency Domain: Individual Alpha Frequency Extraction

To demonstrate the extraction of frequency domain information from the artifact removed EEG data Table 2 shows the IAFs extracted for a single subject in each stimulation case. An increase in IAF is expected when the eyes are closed [40], and this is indeed seen in Table 2. This change is consistent for both the sham conditions and the majority of the stimulation conditions.

### 3.6. ERP Domain: N170 Detection

**Figure 10:**
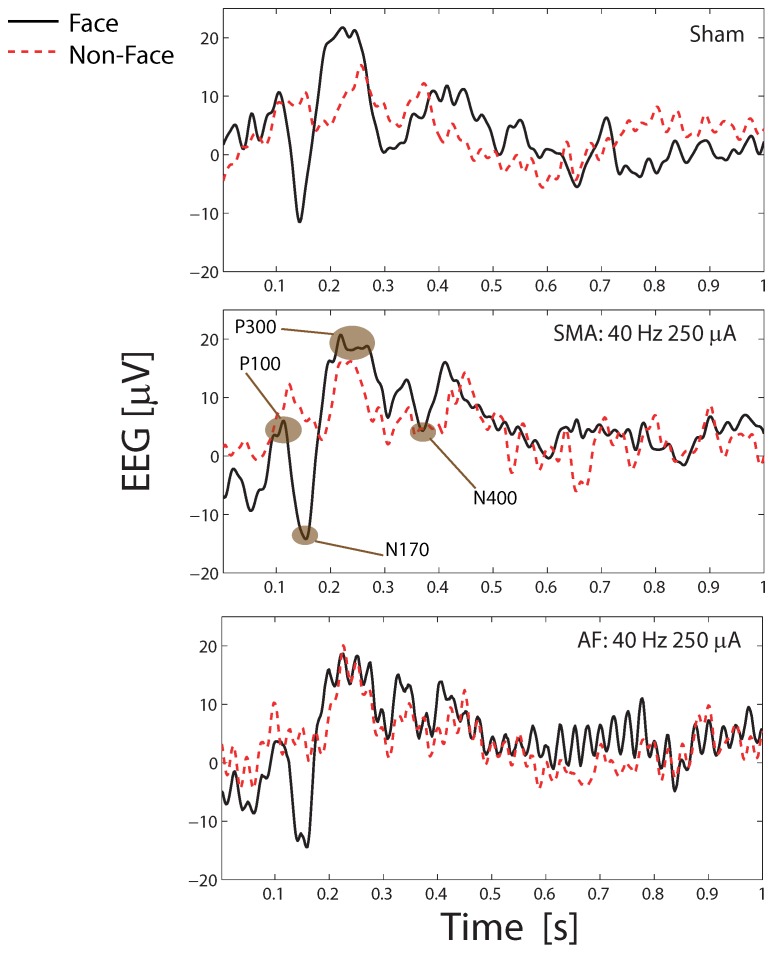
Average ERP at PO8 after application of the detection algorithm for sham and stimulation, at 40 Hz 250 μA stimulation in a single subject. All peaks are detected at the expected times and the expected increases in N170 and N400 depths are seen when face stimuli are presented. Note that the sham and stimulation are different protocols and thus the EEG trace for sham and the other figures are not expected to be identical.

ERP extraction from tACS artifact removed EEG data are illustrated in Figure 10 when using a 40 Hz, 250 μA stimulation. For space here Figure 10 only shows the average ERPs from one subject and stimulation setup. Similar results are found across all subjects and setups. The ERPs are small and below the free-running EEG noise floor signals, giving strong evidence that neural components are correctly reconstructed after artifact removal. The ERP detection accuracy is shown in Table 3 where with all different tACS setups 80% of stimulus presentations result in the detection of a valid evoked response in the EEG. This demonstrates ERP detection even in the presence of tACS stimulation. There is no statistical difference in detection rates (one way ANOVA, p<0.05) between the no stimulation sham case and the artifact removed EEG cases, indicating that ERP trials are not *lost* when tACS is applied.

Figure 10 shows a number of expected evoked peaks: P100 (positive peak 100 ms after stimulus presentation); N170 (negative peak 170 ms after stimulus presentation); and P300 (positive peak between 200 and 400 ms after stimulus presentation). It is clear that despite the simultaneous tACS stimulation a wide number of evoked responses can be correctly recorded and detected in the artifact removed EEG data. This is further verified by the form of the results. When a picture of a face is shown a larger N170 response is expected compared to a non-face presentation [33], and this is indeed seen. An average change in N170 depth of 4.3 μV was seen when a face is presented. Also seen in Figure 10 is the N400 trough, which occurs 300–500 ms post stimuli when the face used is a *famous*/*familiar* one [41], as used in this study. An N400 depth of 7.5–8.5 μV on average was recorded for both sham and stimulation conditions.

Finally, it is noted in Figure 10 that a 40 Hz enveloping is again present after artifact removal when using the AF algorithm. This is similar to the 40 Hz peak in the PSD of Figure 9. It is clear that the AF technique leaves a residual tACS artifact in the EEG signal when a 40 Hz stimulation is used. Nevertheless Figure 10 shows that the low amplitude ERP components can still be easily identified.

## 4. Discussion

There is much debate into the modes of operation, effectiveness and repeatability of transcranial electrical current stimulation [6,7]. This strongly motivates the creation of artifact removal algorithms for EEG to allow simultaneous high temporal resolution monitoring of the brain while it is stimulated. However, to date relatively few studies have systematically developed algorithms for the removal of tACS interference from simultaneously collected EEG recordings. While algorithms are available from the principal tES manufactures (Neuroconn [42] and Neuroelectrics [43]) limited information on their verification methods is available. In the open literature [21] investigated the effect of tACS during sleep using notch filters to remove tACs frequencies, but no performance analysis of this method was presented. Further, the tACS stimulation was at 40 Hz, and so not overlapping with EEG frequency bands of interest for the analysis.

Removing tACS artifacts at stimulation frequencies overlapping with ongoing EEG activity is significantly more challenging. To our knowledge ICA, as used to isolate the tDCS artifact in [18], has not been applied to the tACS artifact removal problem. A method using Principal Component Analysis (PCA) with data recorded from 59 EEG channels during 10 Hz tACS stimulation was developed in [44]. They showed successful removal of the tACS artifact to allow the identification of averaged ERPs in a visual oddball task (P1, N1 and P3 components). As we focus on low channel count EEG systems the PCA [44] based approach cannot be re-applied here for a direct comparison of performance. The application of beamforming filters to remove tACS artifacts in MEG data was investigated in [10,11,45]. This approach was only applied to MEG data in [10,11,45], but the method may be applicable to EEG artifact removal as well. Recently, a new multi-band approach for combining spatial and temporal filtering has been proposed in [46] which is highly applicable for the removal of EEG artifacts. In addition, an open source EEG+tACS toolbox is available from [27] which includes an implementation of the SMA method, but has not yet had performance results published.

In this article we have presented two artifact removal approaches which are independent of the EEG channel count, allowing them to be used with low channel count wearable systems. EEG data after artifact removal has been demonstrated, and verified with no statistically significant differences found when comparing the descriptive statistics of free-running EEG in the time domain, and with ERP extraction being possible, again with no statistically significant differences in extraction rate between sham and artifact removed EEG data. We thus have confidence that true EEG components are being recovered after the artifact removal process and the EEG trace is not dominated by residual artifacts. Undoubtedly however, some residual artifacts remain in on-person testing, seen most noticeably in the 40 Hz stimulation cases when using the adaptive filter. A residual 40 Hz peak is seen in the frequency domain when using this setting, with a 40 Hz oscillation seen in the time domain for both free running and ERP test setups. Nevertheless the presence of such residual artifacts do not prohibit the detection of evoked potentials or the detection of alpha patterns, and only appears at relatively high 40 Hz stimulation settings. It does indicate SMA as the preferred artifact removal approach as the 40 Hz peak is much smaller and not always present, and in everyday usage we believe SMA gives the best practical performance.

The presence of this 40 Hz peak in some artifact removal cases highlights that the verification of on-person artifact removal is intrinsically very challenging as there is no ground truth reference available in order to determine whether any differences in the recovered EEG signal are due to residual or higher order artifacts still being present, or whether they are due to tACS having a modulation effect on the brain and so altering the EEG which is collected, or whether it is because recordings with different stimulation settings cannot be performed simultaneously and so there are simply slightly different EEGs present at different times. As a result there is much debate over *how good* the artifact removal process must be in order to start being of practical use.

To overcome this we have made use of only short duration tACS stimulations (1 min) in order to minimize tACS related changes in the brain. The analysis of longer term (e.g., over a 10–20 min span) changes in the tACS artifact shape and removal performance have not been considered here. We have also introduced a phantom head model to allowed controlled testing of the tACS artifact removal. Similar phantom heads have been used in EEG source localization [47,48], motion artifact characterization [49] and the validation of computation tES modeling [35]. However previous studies in artifact removal (for example [20] for tACS artifacts, and [49] for Transcranial Magnetic Stimulation artifacts) have used melons as head models. The use of a dipole phantom head for amplitude modified tACS artifact characterization was reported in [11], but no details on the structure and composition of the phantom were presented. Our phantom head allows pre-recorded EEG signals to be re-played while the model is stimulated, allowing a quantification of the artifact removal process for the first time as the baseline EEG signal is now known. This shows that SNRs up to 10 dB and high cross correlation coefficients are present for the removal of the gross tACS artifact, helping to build confidence in the methods. We believe that this multi-method approach to artifact removal verification will be critical for building trust that artifact removal methods are obtaining good enough performance to begin to be practically used.

However, our current phantom does not allow more than one EEG source inside the head to be simulated and so the same EEG signal is present at all points on the phantom head. It also does not allow *second order* tACS artifacts to be simulated. Characterizations of the tACS artifact such as [19] have showed that such second order/non-linear artifacts may be present due to “*rhythmic changes of the body’s impedance*” [19] from heart rate and respiration. They suggest that artifact rejection methodologies using PCA, ICA and beamforming filters are not adequate and can result in residual artifacts. In reply, [20] suggest that this effect is not as substantial as initially thought, and that such artifacts may be caused by the stimulator itself, especially when operating at its upper limits (at high impedance/current densities) with the non-linear effects originating at the site of stimulation/recording. During operation a tES stimulator is attempting to maintain a current output. Thus changes in electrode contact impedances result in a change in the voltage output of the stimulator to maintain the constant current and, the resulting artifacts are abruptly changed whenever any significant changes in impedances occur. We see some support for this in our results as from our experience we find that the tACS stimulator’s output impedance is more variable at higher frequencies where our residual artifacts are more likely to be found. Moreover, these 40 Hz frequencies obtained the best correlation coefficients when using the phantom head, which would have a much more stable contact impedance compared to an actual person where respiration, heart beats, micro-movements of the electrodes would be present. For handling such artifacts SMA acts as a comb filter [27], also removing content at the harmonics of the stimulation frequency. Similarly the signal output used with the AF approach in principle records the changes that would arise due to changes in contact impedance and incorporates these into the filter coefficients used for artifact removal at each point in time. Nevertheless such second order and non-linear artifacts are not considered as part of the current study, and we focus only on the removal of the gross tACS artifacts as can be simulated using the current head phantom. In future iterations of head phantom we will seek to investigate whether non-linear artifacts can also be simulated by actively changing the impedance of the head model in a controlled environment.

Finally, our analysis has focused on offline artifact removal algorithms, for data cleaning once all of the EEG data has been collected. For use in real-time applications, which require artifact removal as the data are being collected it is important to comment on the computational complexity present. Both our methods operate using just one channel of EEG data, and can be applied in parallel to the number of channels present. Our implementation on a desktop computer with an Intel i7 processor and 16 GB of RAM takes 0.01 s and 0.04 s for SMA and AF respectively to process 1 s of EEG data. Thus while SMA is quicker, both are highly suitable for real-time use with low channel count EEG systems. Optimizations in the software implementation may be needed to allow real-time use with higher channel count EEG systems. However SMA has a larger requirement on the minimum length of data needed before the reconstructed data are accurate [13]. As *M* cycles of tACS artifacts are averaged to produce an artifact template sufficient time must pass to allow these *M* cycles to occur, and this will depend on the stimulation frequency. Also SMA has a longer convergence time, which on average is observed to be about the length of 1% of the duration of the input data. In contrast, for AF the filter settling time is dependent on the number of filter coefficients used, 64 in this study, giving a quicker convergence time of approximately 60 samples (120 ms) regardless of the stimulation frequency. This comes at the cost of needing to be able to record the output of the tACS stimulator which may not be possible depending on the particular tES product/model used. Between SMA and AF there is thus a trade-off between the artifact removal performance, the time required after the start of tACS stimulation until an artifact removed signal is obtained, and the ease of hardware setup, and this trade-off can be tailored to each different EEG+tACS protocol.

## 5. Conclusions

Transcranial electrical stimulation introduces substantial artifacts into simultaneous EEG recordings which prevent an analysis of the EEG data from during the stimulation. This paper has presented two signal processing algorithms for removing the large scale tACS artifact from simultaneous EEG recordings. Both allow the reconstruction of EEG data which can be visually inspected and allows the extraction of frequency domain components and ERPs during tACS. The methods allow different trade-offs between the artifact removal performance, the time required after the start of tACS stimulation until an artifact removed signal is obtained, and the ease of hardware setup. In addition, a new phantom head model has allowed the quantification of the artifact removal process for the first time with all recovered signal-to-noise ratios being over 6 dB.

## Figures and Tables

**Table 1:** Mean of EEG descriptive statistics: complexity, kurtosis, RMS amplitude, and zero-crossings; calculated using formulas from [39] across all 5 subjects for all 6 repetitions of protocol 1 (n=30 for stimulation data and n=10 for sham data). Also shown are the *p*-values, estimated mean differences and lower and upper Confidence Intervals (CI) from multiple comparisons of sham data with the three reconstruction methods. One way ANOVA results are also included.

Complexity [cbits] − F(3,96) = 1.14, *p* = 0.3370, $\eta^{2}$ = 0.0347					
	Mean	*p*-value	CI: lower limit	Estimated mean difference	CI: upper limit
Sham	6.36	–	–	–	–
SMA	6.95	1.00	−1.62	−0.01	1.60
AF	6.29	0.69	−2.31	−0.69	0.92
Kurtosis [no units] − F(3,96) = 0.36, *p* = 0.7791, $\eta^{2}$ = 0.0114					
	Mean	*p*-value	CI: lower limit	Estimated mean difference	CI: upper limit
Sham	3.80	–	–	–	–
SMA	3.92	0.90	−1.83	−0.38	1.06
AF	3.84	0.95	−1.15	0.30	1.75
RMS [μV] − F(3,96) = 0.99, *p* = 0.4031, $\eta^{2}$ = 0.0302					
	Mean	*p*-value	CI: lower limit	Estimated mean difference	CI: upper limit
Sham	15.56	–	–	–	–
SMA	13.66	1.00	−6.90	−0.13	6.64
AF	15.16	0.97	−5.57	1.22	8.02
Zero crossings [per 10 s/100] − F(3,96) = 0.97, *p* = 0.4080, $\eta^{2}$ = 0.0299					
	Mean	*p*-value	CI: lower limit	Estimated mean difference	CI: upper limit
Sham	12.27	–	–	–	–
SMA	11.85	0.98	−2.46	0.44	3.35
AF	12.53	0.97	−2.41	0.51	3.42

**Table 2:** Individual Alpha Frequency during Eyes Open (EO) stage and Eyes Closed (EC) stage for all protocols and artifact removal algorithms.

Protocol	EO	EC
Sham 1	8.03	8.96
Sham 2	8.07	9.05
SMA: 5 Hz, 0.25 mA	8.03	9.22
SMA: 5 Hz, 1 mA	8.03	9.38
AF: 5 Hz, 0.25 mA	8.03	9.23
AF: 5 Hz, 1 mA	8.03	9.43
SMA: 10 Hz, 0.25 mA	8.03	9.05
SMA: 10 Hz, 1 mA	8.03	9.16
AF: 10 Hz, 0.25 mA	8.03	8.94
AF: 10 Hz, 1 mA	8.03	9.08
SMA: 40 Hz, 0.25 mA	9.19	9.26
SMA: 40 Hz, 1 mA	8.03	9.22
AF: 40 Hz, 0.25 mA	9.40	9.55
AF: 40 Hz, 1 mA	8.03	9.25

**Table 3:** Mean detection scores and standard deviation which represent the percentage of successful trials where an ERP was detected for both face and non-face stimuli. The scores are aggregated for all subjects and all different protocols (n=30 for all stimulation conditions and sham).

Reconstruction Method	Mean Accuracy [%]	Standard Deviation [%]
Sham	84	13
SMA	82	15
AF	86	15
